# Graded spikes differentially signal neurotransmitter input in cerebrospinal fluid contacting neurons of the mouse spinal cord

**DOI:** 10.1016/j.isci.2022.105914

**Published:** 2022-12-30

**Authors:** Emily Johnson, Marilyn Clark, Merve Oncul, Andreea Pantiru, Claudia MacLean, Jim Deuchars, Susan A. Deuchars, Jamie Johnston

**Affiliations:** 1 School of Biomedical Sciences, Faculty of Biological Sciences, University of Leeds, Leeds LS2 9JT, UK

**Keywords:** Molecular neuroscience, Systems neuroscience, Cellular neuroscience

## Abstract

The action potential and its all-or-none nature is fundamental to neural communication. Canonically, the action potential is initiated once voltage-activated Na^+^ channels are activated, and their rapid kinetics of activation and inactivation give rise to the action potential’s all-or-none nature. Here we demonstrate that cerebrospinal fluid contacting neurons (CSFcNs) surrounding the central canal of the mouse spinal cord employ a different strategy. Rather than using voltage-activated Na^+^ channels to generate binary spikes, CSFcNs use two different types of voltage-activated Ca^2+^ channel, enabling spikes of different amplitude. T-type Ca^2+^ channels generate small amplitude spikes, whereas larger amplitude spikes require high voltage-activated Cd^2+^-sensitive Ca^2+^ channels. We demonstrate that these different amplitude spikes can signal input from different transmitter systems; purinergic inputs evoke smaller T-type dependent spikes whereas cholinergic inputs evoke larger spikes that do not rely on T-type channels. Different synaptic inputs to CSFcNs can therefore be signaled by the spike amplitude.

## Highlights

• CSFcN action potentials are mediated by two types of Ca^2+^ channels
• Differential recruitment can generate action potentials of varying amplitude
• CSFcNs can signal their neurotransmitter input with a spike amplitude code
• CSFcNs are mechanosensitive excited by placement of a recording electrode

## Graphical abstract

**Figure:**
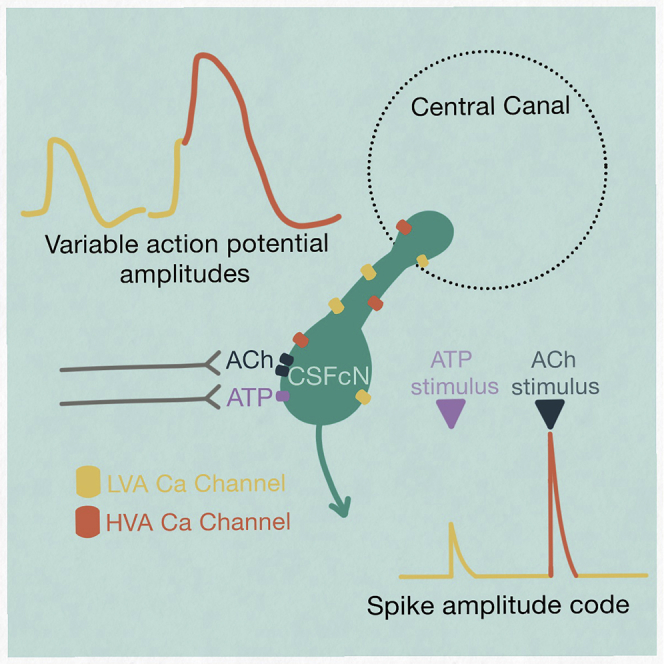

## Introduction

Cerebrospinal fluid contacting neurons (CSFcNs) surround the central canal of the spinal cord in all vertebrate species examined^1^ and possibly in humans.^2^ They project a single dendrite-like structure into the CSF through the ependymal cells that form the border of the central canal. CSFcNs are also present in the caudal medulla oblongata, predominantly surrounding the central canal.^3^^,^^4^

On their identification it was suggested that CSFcNs form a sensory “sagittal organ” within the spinal cord.^5^ This idea is consistent with the observation that CSFcNs are the only cells in the CNS that express polycystic kidney disease 2-like 1 protein (PKD2L1), a channel reported to have chemo- and mechanosensitive properties.^1^^,^^4^^,^^6^^,^^7^^,^^8^ Indeed, studies in zebrafish have indicated that different CSFcN populations respond to bending of the spinal cord and synapse onto distinct motor neuron populations and afferent interneurons to regulate motor behavior. In these studies, disruption of CSFcN signaling leads to impairment of postural control,^9^ spinal morphogenesis,^10^ and locomotion.^9^^,^^11^^,^^12^^,^^13^ Recordings from lamprey also indicate that CSFcNs play a homeostatic role in locomotion; CSFcNs were sensitive to pH and deviations from normal pH reduced locomotor output.^14^^,^^15^ These findings indicate a key role for CSFcNs in spinal sensory signaling in swimming vertebrates and recent work confirms a similar role in quadrupedal locomotion in mice.^16^ The importance of these intriguing cells continues to be realized, yet little is known about the signaling mechanisms of these CSFcNs in mammalian systems.

Voltage-activated Na^+^ channels are widely considered to be fundamental for neuronal excitability and are a requirement for the generation and propagation of action potentials throughout the central and peripheral nervous systems.^17^ Although this assumption holds true in most mammalian neurons, sensory systems commonly utilize Ca^2+^ as the primary mediator of electrogenesis. Within auditory hair cells Ca_V_1.3 (L-type) channels can mediate spikes and glutamate release.^18^^,^^19^ Similarly, retinal bipolar cells rely on low-voltage activated (T-type) Ca^2+^ channels to initiate regenerative potentials and spiking activity.^20^^,^^21^ As voltage activated Ca^2+^ channels operate over a wide range of membrane potentials and facilitate both spiking and graded events, their prevalence enables sensory neurons to respond to a wide range of inputs.^22^^,^^23^ Such a mechanism for signaling would also be advantageous for CSFcNs if they fulfill a sensory role. Single-cell RNA sequencing indicates that mouse spinal CSFcNs abundantly express mRNA for all 3 isoforms of T-type calcium channel (Ca_V_3-1, −2, −3) and numerous high-voltage-activated (HVA) Ca^2+^ channels, including Ca_V_1.3 ^24^ an L-type channel predominantly expressed by sensory neurons and neurosecretory cells.^25^^,^^26^

To begin addressing whether CSFcNs constitute a novel sensory system within the mammalian spinal cord we used 2-photon Ca^2+^ imaging to study the activity of mouse CSFcNs. Our findings reveal that mouse spinal CSFcNs exhibit T-type Ca^2+^ channel dependent spontaneous activity and, in parallel to other sensory systems, employ voltage-activated Ca^2+^ channels to generate spikes with graded amplitudes. CSFcNs can use this amplitude code to signal which of their neurotransmitter systems have been activated.

## Results

### The VGAT promoter drives GCaMP6f expression in all CSFcNs

**Figure 1:**
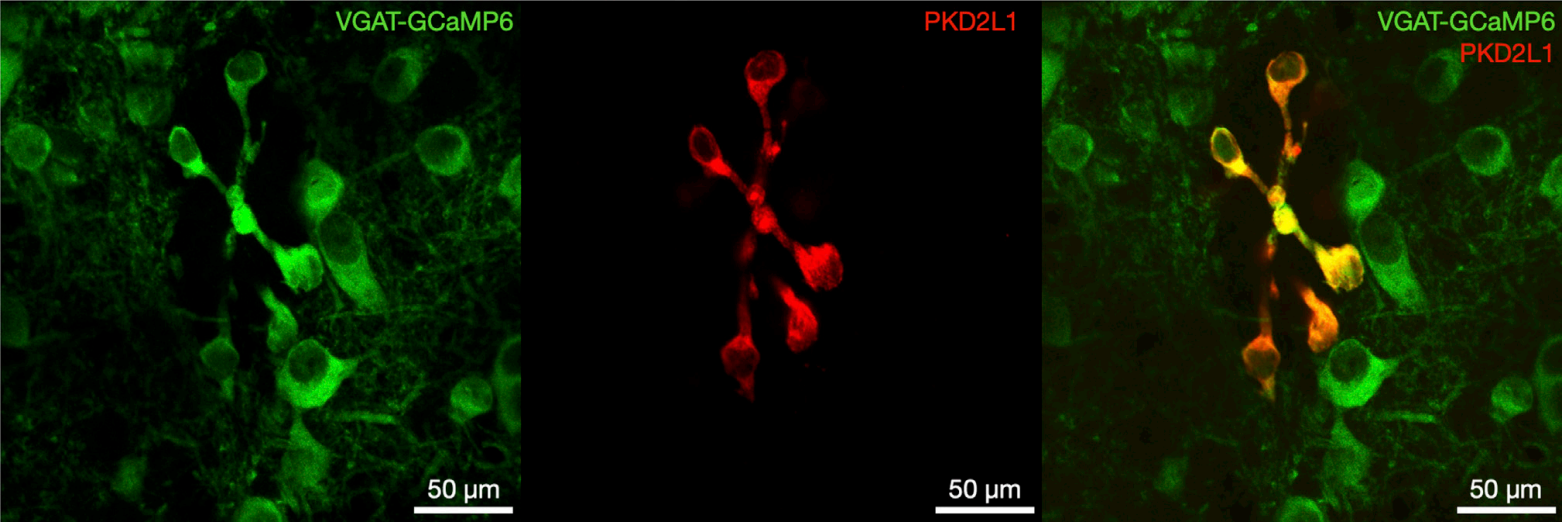
All PKD2L1 expressing CSFcNs are GABAergic left VGAT-GCaMP6 expression around the central canal of the spinal cord. Middle PKD2L1 expression in the same section. Right composite image showing 100% overlap between PKD2L1 and VGAT-GCaMP6. Dorsal side is at the top. Representative data from 6 animals, 12 sections per animal.

To enable imaging of neural activity within CSFcN populations, we targeted GCaMP6f to CSFcNs of the central canal by driving its expression under the VGAT promoter. Within the spinal central canal GCaMP6f was expressed by cells with stereotypical CSFcN morphology, displaying a single bulbous apical process extending into the lumen of the central canal (Figure 1). PKD2L1, the canonical marker of CSFcNs,^1^ displayed 100% overlap with these GCaMP6f positive cells (Figure 1, n= 206, N = 6) and all VGAT+ cells with stereotypical CSFcN morphology were positive for PKD2L1. These data indicate that GCaMP6f is expressed in the entire population of CSFcNs in our VGAT-GCaMP6f mice, and we next took advantage of 2-photon microscopy to image their activity in acute spinal cord slices.

### CSFcNs generate variable amplitude Ca^2+^ spikes insensitive to voltage-activated sodium channel blockade

**Figure 2:**
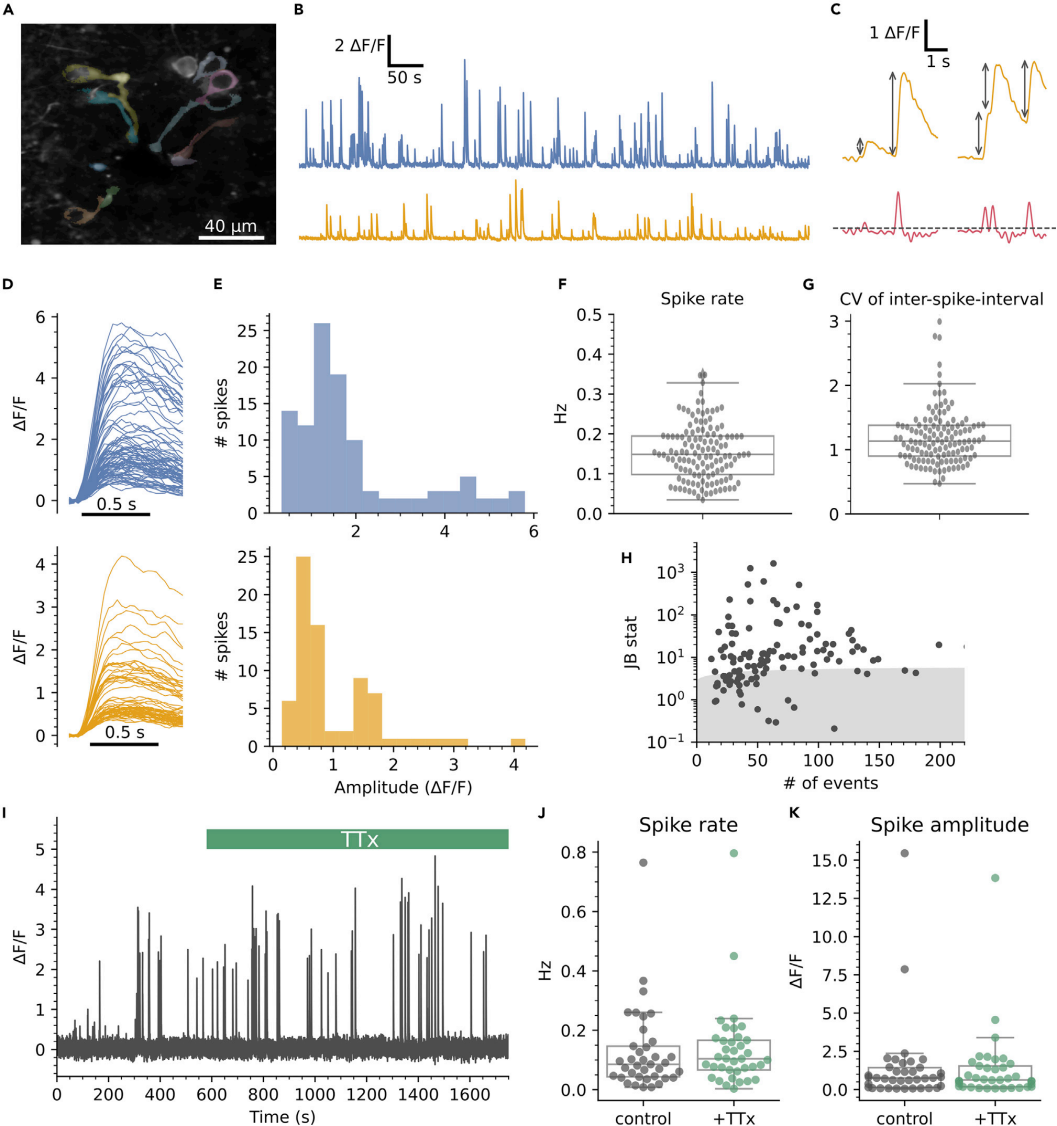
Spontaneous Ca^2+^ spikes in CSFcNs (A) Field of view containing CSFcNs showing segmentation of different CSFcNs. (B) Spontaneous activity of two example cells from A. (C) Spikes were detected on the differentiated trace (red) with an automatically determined threshold (see STAR Methods), gray arrows indicate amplitude measurements. (D and E) The parsed spikes from B, aligned to their onset E Amplitude histograms for the data in D. (F) Spontaneous spike rates from 127 CSFcNs from 15 animals had a median of 0.148 ±0.09 (IQR). (G) The coefficient of variation of the inter-spike intervals across the population was 1.13 ± 0.48 (median ±IQR, n = 127, N = 15). (H) The spike amplitudes within each cell was not normally distributed in 81 of 127 cells, gray shaded area shows critical value of the Jarque-Bera (JB) statistic, see STAR Methods. (I) Ca^2+^ recording of a CSFcN before and during application of 1 μM TTx. (J) TTx had no effect on CSFcN spontaneous spike rate, control 0.085 ±0.1 Hz versus TTx 0.104 ±0.1 Hz, median ±IQR, p = 0.32, Wilcoxon signed rank, n= 37, N = 4. (K) TTx had no effect on the amplitude of spikes, control 0.76 ±1.28 ΔF/F versus +TTx 0.63 ±1.36 ΔF/F, median ±IQR, p = 0.61, Wilcoxon signed rank, n= 37, N = 4. Box plots in F, G, J & K show the median and 25^th^ and 75^th^ percentiles.

We observed widespread spontaneous activity in CSFcNs (Figures 2A and 2B) similar to that reported from *in vivo* Ca^2+^ imaging in the larval zebrafish.^10^ We detected Ca^2+^ spikes using their first derivative (Figure 2C) which enabled separation of summated spikes, see STAR Methods for further details. Spontaneous activity occurred at a low frequency in CSFcNs (Figures 2B and 2F); across a population of 127 CSFcNs the firing rate was 0.148 Hz (median, IQR = 0.097 Hz, n = 127, N = 15). The coefficient of variation for the inter-spike-interval of CSFcNs was close to 1, indicating that their spontaneous activity can be described by a simple Poisson process (Figure 2G). Strikingly, individual CSFcNs could display spikes of variable amplitude, which is illustrated in Figure 2D where the spikes have been aligned by their onset. Such multimodal amplitude distributions were found in 81 of the 127 CSFcNs recorded (Figures 2E and 2H). We presumed that these Ca^2+^ spikes would be the result of spontaneous action potential firing but surprisingly they were insensitive to blockade of voltage-activated Na^+^ channels: 1 μm tetrodotoxin (TTx) had no significant effect on the frequency nor amplitude of spontaneous Ca^2+^ spikes in CSFcNs (Figures 2I–2K). Together these data indicate that CSFcNs display spontaneous Ca^2+^ spikes of variable amplitude that do not depend on voltage-activated Na^+^ channels. This raises the possibility that CSFcNs employ voltage-activated Ca^2+^ channels in place of Na^+^ channels, a phenomenon found in other sensory neurons.^20^^,^^21^^,^^23^^,^^27^^,^^28^

### Variable amplitude Ca^2+^ spikes are due to variable amplitude action potentials

**Figure 3:**
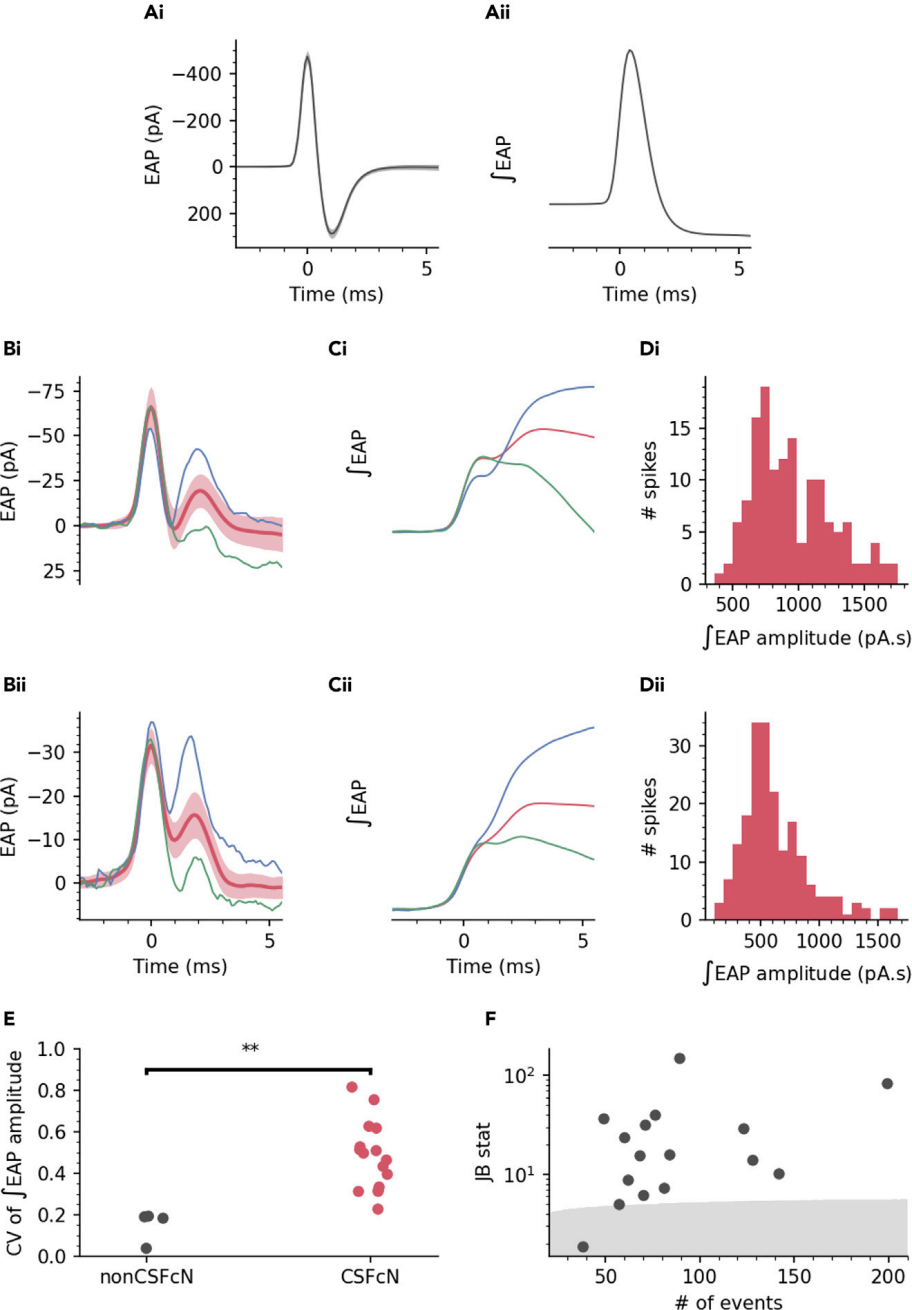
CSFcNs display variable action potential waveforms with dual depolarizing peaks A(i) example of an EAP recorded from a non-CSFcN (mean and SD). (Aii) integral of the mean EAP in Ai. (B) EAPs from two CSFcNs, mean (dark red) and SD (light red) and two single examples of a large (blue) and small (green) 2ndry peak of the EAP. (C) Integrals of the traces shown in B, note the large variation in amplitude. (D) Amplitude histograms calculated for the ∫EAPs shown in C. (E) The coefficient of variation for ∫EAP amplitudes were significantly larger in CSFcNs (n = 16, N = 10) compared to non-CSFcNs (n = 4, N = 4, p = 0.0013, t-test). (F) The ∫EAP amplitudes within each cell were not normally distributed in 15 of 16 cells, gray shaded area shows critical value of the Jarque-Bera (JB) statistic, see STAR Methods.

To explore the nature of the variable amplitude Ca^2+^ spikes in CSFcNs we began by recording extracellular action potentials (EAPs) from identified CSFcNs. EAPs measure the membrane currents associated with the action potential, the integral of which is proportional to the intracellular action potential waveform.^29^^,^^30^^,^^31^^,^^32^^,^^33^^,^^34^ Strikingly, CSFcN action potentials were markedly different from the stereotypical action potential waveform, which is illustrated in Figure 3A. As shown in the two example cells (Figure 3B), CSFcNs displayed two peaks in the depolarizing phase of their EAP indicating that two distinct currents contribute to depolarization of the action potential. Across 16 CSFcNs the median amplitude of the secondary depolarizing current was 23% of the primary with a 25th and 75th percentile of 11 and 37% (n = 16, N = 10). Within a CSFcN the secondary depolarizing current displayed large spike-to-spike variability; it was not evident with every spike (Figure 3B green traces) but could be as large as the initial peak (Figure 3B blue traces). The consequence of a second depolarizing current that is variably recruited is markedly different action potential amplitudes, as illustrated by the integrated EAP waveforms shown in Figure 3C and amplitude distributions in Figure 3D. CSFcNs had significantly more variation in their ∫EAP amplitudes compared to non-CSFcNs (Figure 3E) and similar to the Ca^2+^ spikes shown in Figures 2E and 2H, the amplitude distributions of CSFcN ∫EAPs were multimodal (Figures 3D and 3F). Could these variable amplitude action potentials give rise to the Ca^2+^ spike variability observed in Figure 2?

**Figure 4:**
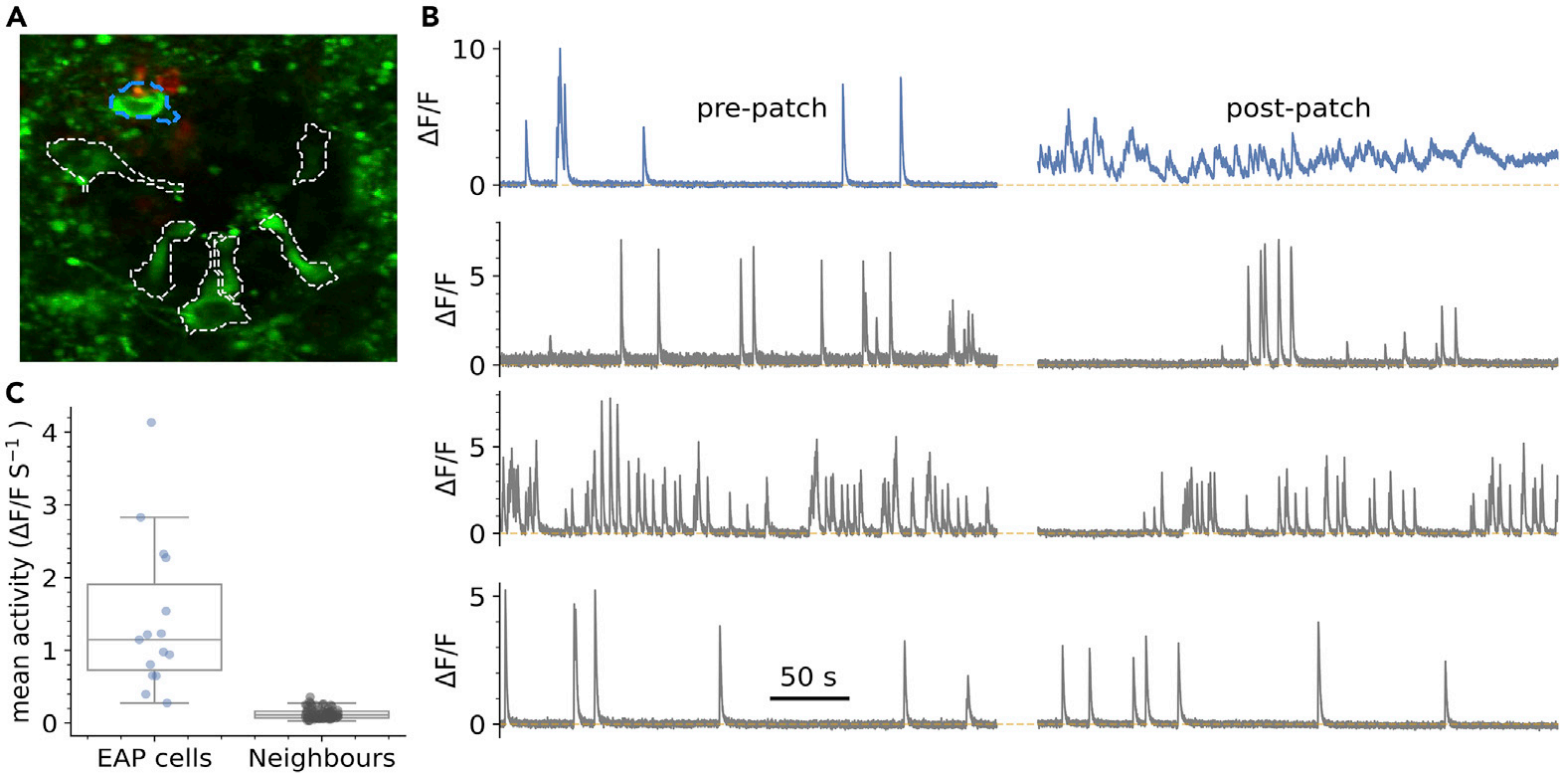
Mechanical activation of CSFcNs via the patch pipette (A) VGAT-GCaMP6 image of CSFcNs (green and dashed outlines) with an EAP electrode against the blue dashed cell. (B) Top: spontaneous activity of the blue cell in A before and after placement of the EAP electrode. Bottom gray rows show 3 neighboring CSFcNs recorded simultaneously in the same field of view that did not have an electrode placed against them. (C) Ca^2+^ activity was higher in all cells with a EAP electrode compare to their neighbors (1.43 ± 1.01 vs 0.13 ±0.7 ΔF/F s-1, p = 1.4 × 10-9, Mann–Whitney U test, EAP n = 15, neighbors n = 66). Box plots in C show the median and 25^th^ and 75^th^ percentiles.

We next sought to correlate the EAP waveform in CSFcNs with simultaneously measured Ca^2+^ spikes. Although the cell-attached recording configuration is minimally invasive, with the intracellular milieu remaining completely unaltered,^32^ we found that placement of an electrode elevated the Ca^2+^ activity in CSFcNs and that this only occurred in the cell against which the EAP electrode was placed. Figure 4 shows the activity of 4 CSFCNs in a field of view, pre and post placement of a patch electrode to record EAPs from the cell in blue. Initially the blue cell displays Ca^2+^ spikes at a typical low rate but once the patch electrode is in place the Ca^2+^ activity remains elevated, despite great care in preventing any depolarization via the pipette;^32^ the other cells in the field of view were unaffected. Higher levels of Ca^2+^ activity was observed with imaging in all 15 cells with simultaneous EAP recordings compared to their neighbors (n= 66, Figure 4C). The spontaneous firing rate was also 3-fold higher when measured with EAPs compared to unperturbed Ca^2+^ imaging (EAP rate 0.43 ±0.18 Hz, mean ±SD, n = 15, N = 10, vs Ca^2^ spike rate: median 0.148 ±0.097 Hz median ±IQR, n = 127, N = 15). We also found that mechanical perturbation of the tissue around a CSFcN evoked activity; pressure pulses applied via a pipette positioned within the tissue and over a CSFcN reliably evoked activity (Figure S1). Whereas changes in the flow of aCSF, either across the tissue (Figure S2) or down the central canal (Figure S3) did not alter CSFcN activity. These observations are consistent with the reported sensitivity of CSFcNs to bending of the spinal cord.^10^^,^^13^^,^^35^

**Figure 5:**
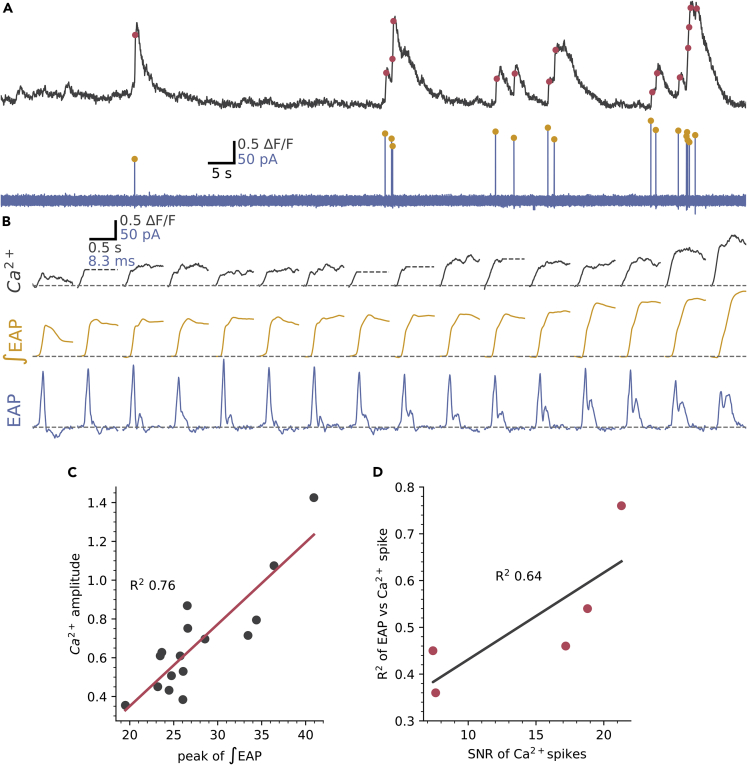
The EAP waveform is correlated with Ca^2+^spike amplitude (A) Simultaneous recording of Ca^2+^ activity (gray) and EAPs (blue). Detected EAPs indicated by yellow dots and Ca^2+^ spikes detected as in Figure 2 are indicated by red dots. (B) The detected spikes from A with expanded time base and ordered by the integral of the EAP. For clarity, Ca^2+^ spikes were truncated (gray dashed line) at the point of a subsequent Ca^2+^ spike. (C) The correlation between the peak of the EAP integral and the Ca^2+^ spike amplitude for the cell in A had a R^2^ of 0.76. (D) The mean R^2^ for the correlation between EAP integral and Ca^2+^ spike amplitude was 0.51 ±0.14 (n = 5, N = 5) and the strength of this relationship depended on the signal-to-noise ratio (SNR) of the Ca^2+^ recordings, see STAR Methods.

Figures 5A–5C shows an example of one of five cells where Ca^2+^ activity was stable enough to allow direct comparison of the EAP and Ca^2+^ spikes. The same spike detection as applied to Figure 2 showed that all detected Ca^2+^ spikes had corresponding EAPs (Figure 5A red dots, Ca^2+^ spike detection). Equally, all EAPs were accompanied by a fast-rising Ca^2+^ spike (Figure 5A, yellow dots, EAP detection). Figure 5B shows the Ca^2+^ spikes expanded and ordered by size with their corresponding EAPs shown below in blue and with the calculated action potential waveforms (∫EAP) in yellow. Similar to Figure 3, the presence of the secondary peak of the EAP varies: when absent the corresponding ∫EAP and Ca^2+^ spike is small (left most panels of Figure 5B), whereas, when the secondary peak is large the ∫EAP and Ca^2+^ spike is large (right side of Figure 5B). Correspondingly, the EAP integral was correlated with the amplitude of the Ca^2+^ spike (Figure 5C), the R^2^ for the cell in Figure 5A was 0.76 and all 5 cells showed a significant correlation with a mean R^2^ of 0.51 ±0.14 (Figure 5D). This shows that a large fraction of the variance in Ca^2+^ spike amplitude is accounted for by differences in the underlying action potential. A significant proportion of the remaining variance is likely explained by measurement noise as, across the 5 cells, the R^2^ value for EAP vs Ca^2+^ spike was correlated with the signal-to-noise ratio (SNR) of the Ca^2+^ spikes (R^2^ = 0.64, Figure 5D). These data indicate that in CSFcNs the action potential and its corresponding Ca^2+^ spike can vary in amplitude depending on the recruitment of a second depolarizing current.

### Two types of voltage-activated Ca^2+^ channels mediate spikes in CSFcNs

**Figure 6:**
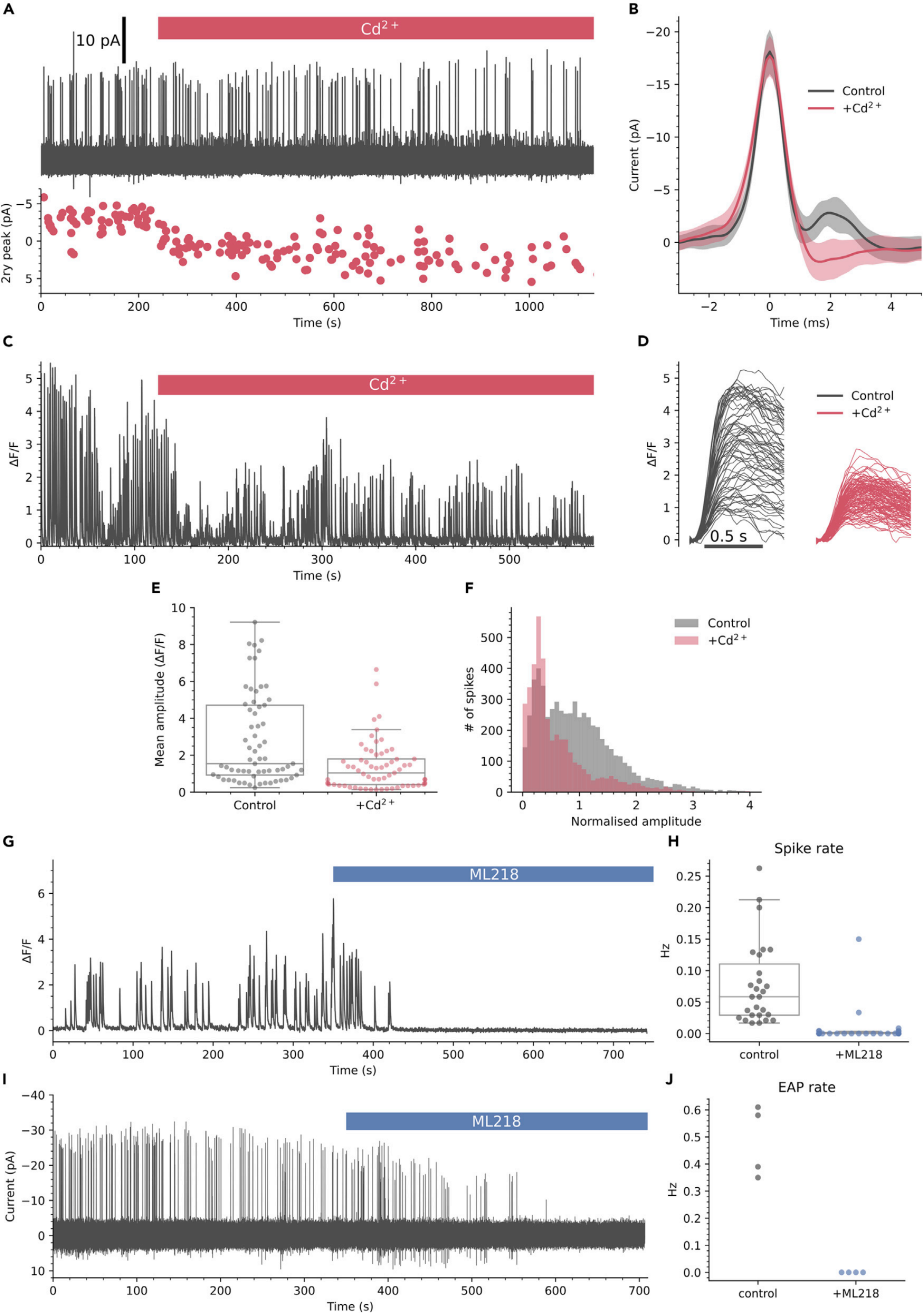
Two types of voltage-activated Ca^2+^ channels mediate different amplitude spikes in CSfcNs (A) EAP recording from a CSFcN during application of 100 μM Cd^2+^ with the amplitude of the 2ndry peak shown below (red dots). (B) The mean ± SD EAP waveform for the first 50 events before Cd^2+^ application (black) and the last 50 events in the presence of Cd^2+^ (red). Cd^2+^ caused a hyperpolarizing shift in the secondary peak of 5.4 ±1.9 pA (n = 4, N = 4). (C) Ca^2+^ spikes recorded from a CSFcN during application of 100 μM Cd^2+^. (D) The Ca^2+^ spikes from C parsed out before (black) and after Cd^2+^ application (red). Note the variable amplitudes in control (black) which become smaller and less variable in Cd^2+^. (E) 100 μM Cd^2+^ significantly reduced the mean amplitude of CSFcN Ca^2+^ spikes (n = 65, N = 5, p = 1.18 × 10^−6^, Wilcoxon signed rank). (F) Histograms of CSFcN amplitudes normalized to their mean amplitude in control. A significant reduction in large amplitude events occurs with Cd^2+^ (n = 65, N = 5, p = 9.33 × 10-15, Kolmogorov-Smirnov test). (G) Ca^2+^ recording from a CSFcN before and during application of 3 μM ML218. (H) ML218 caused a significant reduction in spontaneous firing in CSFcNs, p = 5.6 × 10^−6^, Wilcoxon signed rank test, n = 27, N = 4. (I) EAP recording from a CSFcN before and during application of 3 μM ML218. (J) ML218 caused a significant reduction in spontaneous EAPs in CSFcNs, p = 0.005, n = 4, N = 4. Box plots in E & H show the median and 25^th^ and 75^th^ percentiles.

Similar secondary depolarizing peaks are observed in the EAPs of other slow spiking cells and are due to activation of Cd^2+^ sensitive high voltage-activated (HVA) Ca^2+^ channels.^33^ The variable secondary depolarizing current we observe may therefore be due to differential recruitment of HVA Ca^2+^ channels, a potential source of the amplitude variability of Ca^2+^ spikes. Consistent with this idea 100 μM Cd^2+^, a broad-spectrum HVA Ca^2+^ channel blocker, caused a hyperpolarizing shift in the secondary peak of the EAP in CSFcNs by 5.9 ±2.7 pA (mean ±SD, n = 4, N = 4, Figures 6A and 6B). Correspondingly, Cd^2+^ caused a significant reduction in the mean amplitude of spontaneous Ca^2+^ spikes (Figures 6C–6E). This reduction in the mean amplitude was a result of the larger amplitude spikes being inhibited which can be seen in the single example shown in Figures 6C and 6D and in the normalized amplitude histograms for all spikes from 65 cells (Figure 6F). Together, these data indicate that larger amplitude spikes in CSFcNs are due to recruitment of HVA Ca^2+^ channels (Figures 3, 5, and 6), but what then mediates the smaller Cd^2+^ insensitive spikes? At 100 μM Cd^2+^ effectively blocks all HVA Ca^2+^ channels but has negligible effects on low voltage-activated (LVA) T-type Ca^2+^ channels.^36^ Therefore, we tested whether T-type Ca^2+^ channels were responsible for the smaller amplitude Ca^2+^ spikes. The selective T-type Ca^2+^ channel blocker^37^ ML218 (3 μM) dramatically reduced spontaneous Ca^2+^ spikes in CSFcNs (Figures 6G and 6H), indicating that the smaller Ca^2+^ spikes require T-type Ca^2+^ channels and, without these smaller spikes, HVA Ca^2+^ spikes do not get recruited. Consistent with this, ML218 also decimated spontaneous EAPs in all CSFcNs tested (Figures 6I and 6J), showing that the initial peak in the EAP is mediated by T-type Ca^2+^ channels. Together these data imply that spontaneous firing in CSFcNs requires T-type Ca^2+^ channels and these mediate the initial phase of depolarization, then if a threshold is reached, HVA Ca^2+^ channels are recruited to boost the amplitude of the action potential and Ca^2+^ spike.

### Spike amplitude as a signal for neurotransmitter activation

**Figure 7:**
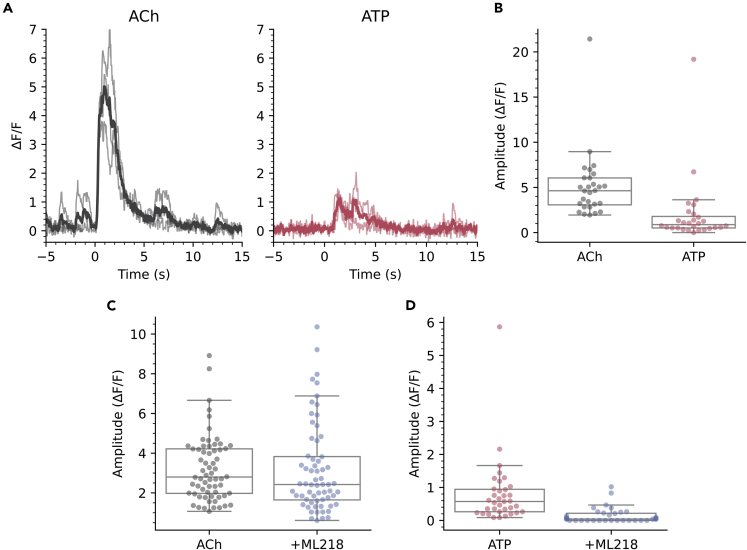
CSFcNs signal acetylcholine and ATP inputs with different amplitude spikes (A) The response of a CSFcN to focal application of Acetylcholine (Ach 1 mM, black traces), the same CSFcN responding to focal application of ATP (300 μM, red traces). 3 trials are overlain with the average shown in dark. Experiment was conducted in the presence of 20 μM NBQX, 50 μM APV, 10 μM GABAzine, 1 μM strychnine and 1 μM atropine. (B) CSFcNs generated larger spikes to ACh (4.63 ΔF/F IQR 2.98) compared to ATP (0.88 ΔF/F IQR 1.28, n = 28, N = 3, p = 4.716 × 10-6, Wilcoxon signed rank. (C) 3 μM ML218 had minimal effect on Ach evoked responses, 2.80 ΔF/F IQR 2.24 vs 2.42 ΔF/F IQR 2.18, n = 64, N = 3, p = 0.536, Wilcoxon signed rank. (D) ML218 caused a significant reduction in ATP evoked responses, 0.57 ΔF/F IQR 0.68 vs ATP + ML218: 0.04 ΔF/F IQR 0.22, n = 35, N = 4. Box plots in B-D show the median and 25^th^ and 75^th^ percentiles.

How might CSFcNs use these graded amplitude spikes? We reasoned that CSFcNs may be able to generate different amplitude spikes depending on their synaptic input; those inputs providing only weak depolarization may generate small amplitude T-type dependent spikes, whereas inputs providing robust depolarization could directly recruit the larger HVA Ca^2+^ spikes. The somatic membranes of CSFcNs express both nicotinic^38^ and P2X receptors.^39^ To test whether CSFcNs can use their spike amplitude to distinguish between these different neurotransmitter systems we stimulated CSFcNs with focal ejection of saturating concentrations of each agonist: 1 mM acetylcholine and 300 μM ATP (Figures 7A and 7B).^40^^,^^41^ Within the same CSFcN acetylcholine generated larger spikes than ATP (Figures 7A and 7B). These experiments were conducted in the presence of a cocktail of synaptic antagonists (20 μM NBQX, 50 μM APV, 10 μM GABAzine, 1 μM strychnine, 1 μM atropine) to prevent any recurrent excitation via non-CSFcNs responding to the agonists. The synaptic blocker cocktail did not affect the amplitude of response suggesting the agonists act directly on CSFcNs and not through excitation of presynaptic neurons (ACh: 4.52 ΔF/F ±3.03 vs ACH + synaptic blockers: 4.63 ΔF/F ±2.98, p =0.716, ATP: 1.51 ΔF/F ±2.16 vs ATP + synaptic blockers: 0.88 ΔF/F ±1.28, n = 28, N = 3, p = 0.151, Wilcoxon signed rank). Standard aCSF focally ejected in place of the agonists did not evoke activity (Figure S3), indicating no contribution from mechanical activation at the pressures used for drug application. These data indicate that CSFcNs can use an amplitude code to signal whether cholinergic or purinergic inputs have been activated. Furthermore, we found that block of T-type channels with ML218 almost eradicated the response of CSFcNs to ATP (Figure 7D) but had less of an effect on acetylcholine evoked spikes (Figure 7C). It seems that CSFcNs respond to purinergic inputs with only small amplitude T-type dependent spikes, whereas cholinergic inputs provide sufficient drive to evoke larger amplitude spikes without the need of initial depolarization through T-type Ca^2+^ channels.

## Discussion

The spike, or action potential, and its “all-or-none” nature is thought to be a fundamental quantum of neural processing. We demonstrate that rather than the typical voltage-activated Na^+^ channels, CSFcNs in the spinal cord of mice use voltage-activated Ca^2+^ channels to generate spikes and that by employing different types of Ca^2+^ channel they can generate graded spikes of variable amplitude. CSFcNs can use these graded spikes to differentially signal which neurotransmitter system is providing their input.

Na^+^ dependent spikes are the ubiquitous mechanism for action potential generation throughout the mammalian brain,^42^ whereas Ca^2+^ spikes are typically utilized for dendritic signal integration.^43^^,^^44^^,^^45^ It seems that the somas of CSFcNs can perform similar signal integration to that normally assigned to dendrites, distinguishing between different synaptic inputs. Both our EAP and Ca^2+^ imaging recordings provide localized measurements of activity, recording the somatic membrane currents associated with the action potential and the Ca^2+^ activity within the soma and endbulb respectively.^30^^,^^31^ This was advantageous for our purpose of observing the correlation between electrical and Ca^2+^ spikes (Figure 5), yet it does not preclude a role for Na^+^ spikes further down the axon of CSFcNs, which project as far as the ventral fissure,^39^ but it does suggest that Na^+^ channels are absent or at low densities in the soma and endbulb.

In a close parallel, somatic Ca^2+^ spikes are a prominent feature of various other sensory cells including retinal bipolar cells^23^ and auditory hair cells.^27^ Retinal bipolar cells signal with graded analog signals as well as spikes,^21^ both of which are mediated by voltage-activated Ca^2+^ channels.^20^^,^^28^ Such variations in the amplitudes of Ca^2+^ signals likely support the multivesicular amplitude code used by these synapses.^46^^,^^47^ Both T-type and L-type Ca^2+^ channels contribute to spiking in retinal bipolar cells^20^ and in a direct parallel with our findings, T-type channels are required for spontaneous firing whereas L-type channels influence the shape and duration of Ca^2+^ events.^48^ During development inner hair cells of the cochlea can also signal with graded and spiking responses predominantly through voltage-activated Ca^2+^ channels^27^^,^^49^ and spontaneous Ca^2+^-mediated action potentials in hair cells are intrinsically generated and influenced by both ACh and ATP.^49^ Since their identification, CSF-CNs have been proposed to be sensory neurons^5^ and more recent work has demonstrated their functional role in both mechano- and chemo-sensation.^9^^,^^12^^,^^13^^,^^50^ It seems then that the adoption of Ca^2+^ spikes to enable variable amplitude Ca^2+^ events is a common feature of neurons across different sensory systems.

Our data indicate that spontaneous firing at the soma of CSFcNs is dependent on T-type Ca^2+^ channels (Figure 6), this occurs with a low mean rate of ∼1 spike every 10 s and rather than being rhythmic, the CV of ∼1 for the inters-spike-interval implies spontaneous activity occurs because of stochastic processes (Figure 2). Of interest, the spontaneous rate we measured with imaging alone, (i.e., without an electrode) is identical to that measured with an electrode in a previous study but from CSFcNs lacking PKD2L1.^9^ We demonstrate that CSFcN activity is altered by placement of an electrode against a CSFcN (Figure 4) or by deformation of the tissue with pressure pulses applied above a CSFcN (Figure S1), a prerequisite step to forming a seal with a patch electrode. The spontaneous rate we measured with a patch electrode is identical to previous electrical recordings from CSFcNs with PKD2L1 intact.^8^ It seems then that mammalian CSFcNs are mechanosensitive like their zebrafish and lamprey counterparts^10^^,^^13^^,^^50^ and that PKD2L1 likely plays a role in this sensitivity. The PKD family of genes are thought to contribute to mechanosensation,^51^ in particular, sensing the shear stress of flow in kidney cells^52^ and movement of the CSF in CSFcNs,^10^ they may also sense the viscosity of the extracellular matrix.^53^ Mechanical perturbation of the membrane of CSFcNs clearly alters the basal firing state of CSFcNs (Figures 4 and S1) and this may be because of increased activity of PKD2L1, indeed previous electrical recordings have shown that even a single opening of a PKD2L1 channel is sufficient to evoke action potential firing in CSFcNs.^8^ This highlights an advantage of optical methods for measuring the activity of putative mechanosensitive cells.

Recent work implicates CSFcNs in adaptive motor control for skilled movements in the mouse,^16^ yet how these neurons integrate information has not yet been fully determined. For example, they are recipients of numerous axon terminals on their basal pole; including purinergic and cholinergic inputs^38^^,^^39^ and possibly serotonergic.^54^ We show that inputs from purinergic and cholinergic receptors are processed differently by CSFcNs; purinergic inputs are weaker, evoking smaller amplitude T-type dependent Ca^2+^ spikes whereas cholinergic inputs evoke larger spikes that do not depend on T-type channels (Figure 7). The source of the cholinergic inputs is most likely to be spinal in origin because CSFcNs maintained in long-term spinal cord cultures respond to positive allosteric modulators of nicotinc Ach receptors.^38^ Ideal candidates that could provide these inputs are the nearby cluster or cholinergic partition interneurons in lamina X.^55^ Similar to other regions of the central nervous system, purinergic inputs are harder to pin down. ATP can be coreleased from many different synaptic terminals that carry other neurotransmitters and can be released independent of other cargo.^56^^,^^57^ ATP release can also be evoked from spinal cord astrocytes in response to synaptic inputs.^58^ ATP is also released in response to mechanical stimulation, a change in pH and may also be part of damage signaling mechanisms,^59^ fitting well with the idea that CSFcNs are sensory cells.

A pertinent question is what role the different amplitude spikes in CSFcNs play? The apical process within the lumen of the central canal contains vesicles^60^^,^^61^ and likely releases GABA from this site; in lamprey, CSFcNs containing somatostatin dense core vesicles appear to release these from their endbulb/soma.^62^ How might the different spike types we describe contribute to transmitter release? Typically, the HVA P/Q and N-type Ca^2+^ channels are coupled to transmitter release in neural cells and N-type channels are present in CSFcNs.^63^ However, numerous examples of T-type Ca^2+^ channel activity governing transmitter release exist, including in: neuroendocrine cells in the pituitary,^64^ adrenal glands,^65^ retinal bipolar cells^66^ and olfactory bulb neurons.^67^^,^^68^ It is therefore likely that the smaller T-type Ca^2+^ spikes that we observe in CSFcNs are able to evoke release of vesicles, perhaps with the HVA channels providing higher vesicle release rates or release of dense-core vesicles. Such dual modes of release have been described in chick auditory hair cells where T-type Ca^2+^ currents regulate rapid vesicle release and L-type Ca^2+^ channels regulate sustained neurotransmitter release.^69^ Furthermore, the N-type Ca^2+^ channel in medullar CSFcNs can be modulated by GABA_B_ receptors,^63^ which provides further means for CSFcNs to modulate their spike properties dependent on their synaptic input. In addition to releasing transmitter from their endbulb, CSFcNs also send an axon to the ventromedial fissure where their synaptic terminals intermingle with axons of the corticospinal tract and short-range projections to spinal interneurons.^11^^,^^13^^,^^16^^,^^39^ The different amplitude spikes we describe may offer the ability to multiplex output signals in a target dependent manner; possibly only the larger spikes propagate as far as the corticospinal tract, whereas both large and small spikes influence local interneurons and release from the endbulb. Simultaneous imaging of Ca^2+^ spikes with transmitter release would shed light on these intriguing possibilities, but would require use of a spectral variant of GCaMP^70^ and improvements to the fluorescent reporter of GABA to enable monitoring of individual synapses.^71^

In conclusion, we provide novel evidence that CSFcNs use T-type Ca^2+^ channels to generate spontaneous activity and to respond to low amplitude inputs. We reveal how CSFcNs can use graded Ca^2+^ spikes to respond to inputs from different neurotransmitter systems via distinct mechanisms. These observations closely mirror findings within other sensory systems and are consistent with CSFcNs functioning as a multimodal sensory neuron within the mammalian spinal cord.

### Limitations of the study

Our recordings were only made between T5–L3 of the spinal cord, as CSFcNs line the entire spinal cord and ventricles we cannot be certain our finding generalize to CSFcNs in the sacral chord or ventricles. We describe action potential properties of CSFcNs (Figure 3), but we also show that mechanical perturbation with a patch electrode elevates the activity of CSFcNs (Figures 4 and S1). The spontaneous firing rate that we report for CSFcNs measured with an electrode is therefore likely to be elevated above their basal level. These findings illustrate a benefit of Ca^2+^ imaging to monitor mechanosensitive cells; one can avoid inadvertently stimulating them with the recording apparatus. Finally, although we demonstrate that CSFcNs can generate distinct spike amplitudes for different synaptic inputs, further work is required to identify the behaviors and circuits that drives synaptic activity onto CSFcNs and how the different amplitude spikes influence spinal circuitry.

## STAR★Methods

### Key resources table

**Table:** 

REAGENT or RESOURCE	SOURCE	IDENTIFIER
**Antibodies**		

Rabbit polyclonal PKD2L1	Proteintech	13117-2-AP
Chicken polyclonal GFP	Abcam	ab13970
Alexa Fluor 488 anti-chicken	ThermoFisher	A32931
Alexa Fluor 555 anti-rabbit	ThermoFisher	A-31572

**Chemicals, peptides, and recombinant proteins**		

ML218	Sigma-Aldrich	Cat: SML0385
Cadmium Chloride	Sigma-Aldrich	Cat: 202908
Acetylcholine chloride	Sigma-Aldrich	Cat: A6625
ATP	Sigma-Aldrich	Cat: A1852
Gabazine	Sigma-Aldrich	Cat: S106
Strychnine hydrochloride	Sigma-Aldrich	Cat: S8753
Atropine	Sigma-Aldrich	Cat: A0132
NBQX	Sigma-Aldrich	Cat: N183
APV	Sigma-Aldrich	Cat: A5282

**Experimental models: Organisms/strains**		

VGAT-ires-cre	Jackson Laboratories; H. Zeng	RRID:IMSR_JAX:028862
Floxed-GCaMP6f	Jackson Laboratories; B. Lowell	RRID:IMSR_JAX:028865

**Software and algorithms**		

Igor Pro	Version 8	https://www.wavemetrics.com
Neuromatic	Version 3	http://www.neuromatic.thinkrandom.com
Python	Python Software Foundation	https://www.python.org
Suite2p	v0.10.1	https://github.com/MouseLand/suite2p
Matlab	Matlab R2020b	https://www.mathworks.com
Scanimage	V5	https://vidriotechnologies.com
FIJI	Image j	https://imagej.net/software/fiji/

### Resource availability

#### Lead contact

Further information and requests for resources and reagents should be directed to and will be fulfilled by the lead contact, Jamie Johnston (j.johnston@leeds.ac.uk).

#### Materials availability

This study did not generate new unique reagents.

### Experimental model and subject details

#### Animals

Animal handling and experimentation was carried out according to UK Home Office guidelines and the requirements of the United Kingdom Animals (Scientific Procedures) Act 1986 and the University of Leeds animal welfare ethical review board. Mice were housed under a 12:12 h light/dark cycle with free access to food and water. All efforts were made to minimize animal suffering and the number of animals used. Vesicular GABA transporter-IRES-Cre mice (*VGAT.Cre*, stock 028,862, B6J.129S6(FVB)-Slc32a1<tm2(cre)) were crossed with floxed GCaMP6f mice (*GCaMP6f.flox,* stock 028,865, B6J.CgGt(ROSA)26Sor < tm95.1 (CAGGCaMP6f)), to generate VGATxGCaMP6f mice. Both mouse lines were originally from Jackson Laboratory (Maine, USA) and maintained in house. Consistent with the NC3Rs guidelines (https://www.nc3rs.org.uk/who-we-are/3rs), both males and females aged P19-52 were used in this study.

### Method details

#### Immunohistochemistry

3 VGATxGCamP6f mice at P18 and 1 each at P30, P46 and P52 were anesthetized with a terminal dose of sodium pentobarbital (100 mg.kg-,^1^ I.P, Euthatal, Merial Animal Health) and transcardially perfused, initially with phosphate buffer (PB, 0.1M) to remove blood, and then with paraformaldehyde (PFA, 4% in 0.1M PB, 250 mL). Brains and spinal cords were removed and post-fixed overnight in PFA. Spinal cords were serially sectioned at 40 μm with a vibrating microtome (VT1200, Leica Microsystems) and stored in PBS at 4⁰C. Sections were incubated with anti-PKD2L1 (1:500, rabbit, Proteintech) and anti-GFP (1:1000, chicken, Abcam) dissolved in PBS with 0.2% Triton X-100 with 5% donkey serum as a non-specific binding blocker. Sections were washed (x3, PBS 10 min) before addition of the Alexa Fluor conjugated secondary antibody (1:1000 in PBS, Thermofisher) at room temperature for 2h. Sections were washed (x2 PBS, 10 min) before being mounted on microscope slides and allowed to air dry. Sections were covered using vectashield with DAPI (VectorLabs, cat no. H-1800) and a coverslip was added and sealed using nail varnish.

#### Acute slice preparation

VGATxGCamP6f mice (P19-P47, both sexes) were terminally anesthetized with sodium pentobarbital (as above) and decapitated. The spinal column was dissected to enable access to both cut ends of the vertebral column. A 25 mL syringe attached to an 18 G needle and filled with oxygenated (95% O_2_: 5% CO_2_) sucrose artificial cerebrospinal fluid (sucrose-aCSF, 30°C, 26 mM NaHCO_3_, 2.5 mM NaH_2_PO_4_, 3 mM KCl, 217 mM sucrose, 10 mM glucose, 2 mM MgSO_4._7H_2_0, 1 mM CaCl_2_) was placed into the caudal end of the spinal canal. Manual pressure was then applied to the caudal end of the cord to gently eject the entire cord through the rostral cut end of the vertebral column.^72^ Thoracolumbar spinal cord (T5-L3) was embedded in agar (1.5% in sucrose-aCSF) and transverse section 400 μm thick were collected using a vibrating microtome (Integraslice 7550, Campden Instruments), in sucrose-aCSF (30°C). Spinal cord sections were transferred to a submerged incubation chamber containing standard aCSF (124 mM NaCl, 26 mM NaHCO_3_, 10 mM glucose, 3 mM KCl, 2.5 mM NaH_2_PO_4_, 2 mM MgSO_4._7H_2_0, 2 mM CaCl_2_, room temp), for ≥1 h prior to recording.

#### 2-Photon Ca^2+^ imaging

Spinal cord slices were transferred to the recording chamber, of a custom built 2-photon laser scanning microscope, and perfused with oxygenated aCSF (room temp, 20 mL/min), driven by a peristaltic pump. GCaMP6f fluorescence was excited at 910 nm using a pulsed Mai Tai eHP DeepSee Ti:sapphire laser system (SpectraPhysics). A resonant-galvo mirror assembly (Sutter instruments) scanned the beam through a 16× water-dipping objective (N16XLWD-PF, NA 0.8, Nikon). Fluorescence was detected using GAasP photomultiplier tubes and appropriate filters and dichroic mirrors. Images were acquired at 30-120Hz, using ScanImage v.5 software.^73^ Focal ejection of agonists was achieved using microinjection patch electrodes (3-4 μm tip diameter at 2-4 psi) connected to a picospritzer II (Parker) and positioned above the central canal. Agonist were dissolved in aCSF and 12.5 μM Sulforhodamine 101 was included to visually confirm drug delivery. aCSF delivery alone did not alter CSFcN activity (see Figure S3).

#### Extracellular action potential recordings

Cell attached recordings were made using an axopatch 200B (Molecular Devices) signals where digitised using an NI-6356 A-D converter (National Instruments) controlled by Neuromatic software,^74^ which runs in Igor Pro (Wavemetrics). Recordings were made with patch pipettes (3-5 MΩ) containing aCSF and 10 μM Alexa 594 (Thermofisher) and CSFcNs were targeted under visual guidance using their fluorescence. Light suction was applied to form a seal (10-200 MΩ) and extracellular action potentials (EAPs) were recorded in voltage-clamp mode with the command voltage set to give 0 current. This configuration, 0 current through the pipette and a loose seal, is the optimum for ensuring no depolarisation of the cell being recorded.^32^ EAPs are shown inverted so that depolarising phase is rising and hyperpolarising phases are falling.

*Drugs and chemicals:* All chemicals and drugs were purchased from Sigma-Aldrich unless otherwise stated.

### Quantification and statistical analysis

#### Immunohistochemical analysis

For each section a z stack was taken with a LSM880 confocal (Zeiss) to capture both the soma and endbulbs of CSFcNs. Using FIJI^75^ CSFcNs were manually counted in the maximum intensity projection and were identified in the VGAT-GCaMP6 channel as cells possessing a single bulbous apical process extending into the central canal.

#### Image segmentation of CSFcNs

The suite2p pipeline v0.10.1 ^76^ was used to extract raw fluorescence time courses from CSFcNs. Data was first registered using the default options (‘nimg_init’: 200, ‘batch_size’: 200, ‘maxregshift’: 0.1, ‘smooth-sigma’: 1.15). ROI detection was then performed using a spatial scale of 24-48 pixels. The ROI masks corresponding to CSFcNs where visually checked and non-CSFcNs, defined as cells lacking a single bulbous apical process extending into the central canal, were excluded from further analysis. Suite2p and related algorithms rely on sparse asynchronous neural activity to segment cells and we found this approach worked well for the spontaneous spiking data shown in Figures 2, 4, 5, and 6. However, for the data in Figure 7, the focal application of agonists generated synchronous activity across many CSFcNs, we therefore extracted fluorescent time courses for these experiments by manually drawing the cells in FIJI.^75^ Extracted fluorescent traces were normalised as ΔF/F using the following equation: F-F^0^/F^0^, where F is the raw florescent trace and F^0^ is the baseline fluorescence which we defined as the 15^th^ percentile of the raw fluorescence.

#### Spike detection

Ca^2+^ spikes were detected using the differentiated fluorescence signal as shown in Figure 2C. The fluorescence trace was first filtered with a third order Savitzky-Golay filter with a 300 ms window. This signal was then differentiated and point-to-point fluctuations were supressed with a 5 point median filter and then filtered with a third order Savitzky-Golay filter with a 166 ms window. SciPy’s ‘signal.find_peaks’ function was then used to detect spikes with a threshold of 5 times the median-absolute-difference of the differentiated signal. EAPs were detected on the band-pass filtered signal (6-1500 Hz), again using ‘signal.find_peaks’ with the threshold set independently for each cell.

#### Statistical analysis

Ca^2+^ spike amplitudes were measured as the difference between the peak ΔF/F signal occurring in a 333ms window around the spike time and the mean signal in the preceding 100ms (gray arrows in Figure 2C). To capture the multi-modality of the amplitude distributions for CSFcNs we used the Jarque-Berra statistic (Figures 2F and 3F) and plot the critical value for a significance level of 0.05 (shaded area) obtained from the Jarque-Bera simulation in Igor Pro. If all data were normally distributed only 5% of points would be outside the shaded area. For calculation of the relative amplitude of the EAP’s secondary depolarising current we took the mean EAP waveform and found the maximum value after the third inflection point of the EAP’s differential (which corresponds to the end of the initial repolarisation phase). This accounted for the variable delay in the time of the secondary peak. The SNR of Ca^2+^ spikes in Figure 5 was calculated by dividing the spike amplitudes by the SD of the fluorescence measured in the 180 ms preceding each spike. The mean SNR across all spikes was used for plotting in Figure 5D. To prevent cell-to-cell variation in spike amplitude biasing the spike distributions in Figure 6F, the amplitudes measured in control and with Cd^2+^ were both normalised by the mean amplitude of each cell in control. For graphs of summary metrics individual cells are shown as round markers and boxplots represent the median, 25^th^ and 75^th^ percentiles, whiskers extend by 1.5 x interquartile range. All stated summary statistics are either mean ± SD or median ± interquartile range as appropriate and stated in the text. ‘n’ is used to represent a single cell and ‘N’ is used to represent an animal. Where significant differences are reported the statistical test is stated after the p value.

## Data Availability

• All data reported in this paper will be shared by the lead contact upon request. • This paper does not report original code. • Any additional information required to reanalyse the data reported in this paper is available from the lead contact upon request.
