# Supplementary material for: Graded spikes differentially signal neurotransmitter input in cerebrospinal fluid contacting neurons of the mouse spinal cord
Source: iScience. 2022 Dec 30;26(1):105914. doi: 10.1016/j.isci.2022.105914 (PMC9860393; doi:10.1016/j.isci.2022.105914)
Supplement: Document S1. Figures S1–S3 [file mmc1.pdf]

iScience, Volume *26*

Supplemental information

Graded spikes differentially signal neurotransmitter input in cerebrospinal fluid contacting neurons of the mouse spinal cord

Emily Johnson, Marilyn Clark, Merve Oncul, Andreea Pantiru, Claudia MacLean, Jim Deuchars, Susan A. Deuchars, and Jamie Johnston

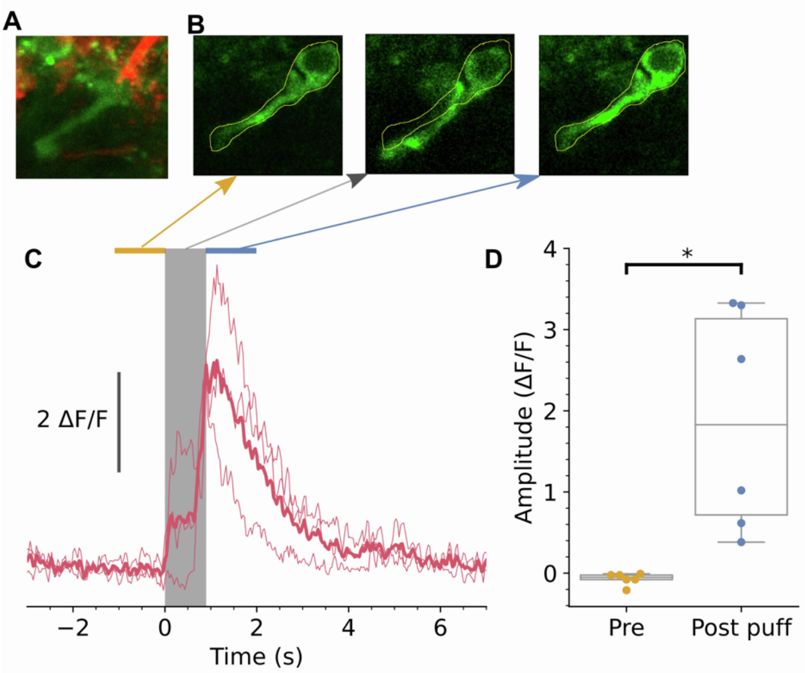

Figure S1: Deformation of the tissue activates CSFcNs, related to Figure 4 and STAR Methods. **A** A puffing electrode placed in the tissue above a CSFcN. **B** The CSFcN immediately before a 10 psi puff, during the puff and immediately after. Yellow, outline indicates cell location before puff. Note the displacement of the cell during the puff which returns to the same location after the puff. **C** Response of the cell in B to 3 trials (light colour) with the mean (dark). **D** The mean ΔF/F over 3 trails and during 1 s after the pulse (blue bar in C) was significantly greater than the mean ΔF/F preceding the pressure puff (yellow bar in C), p=0.018, paired t-test, n=6, N=3.

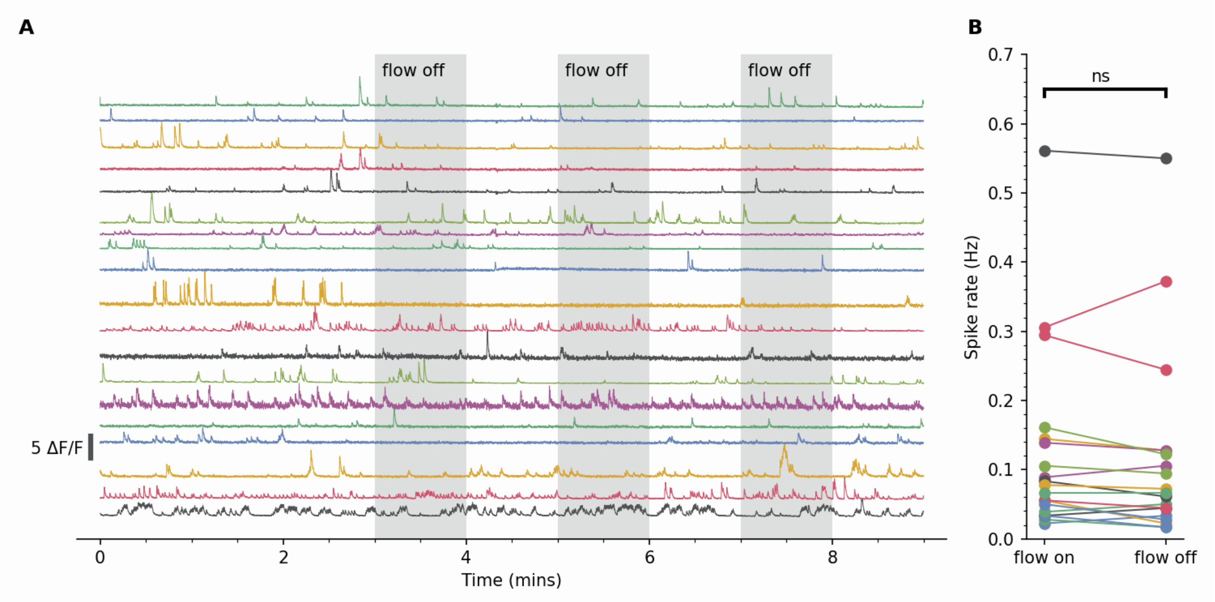

Figure S2 aCSF flow over the tissue does not modulate CSFcN activity, related to Figure 4 and STAR Methods. A Spontaneous activity from 19 CSFcNs from 3 animals before and during 3 x 1 minute periods where aCSF perfusion was halted (shaded areas). **B** The spike rate during aCSF flow on vs flow off was not significantly different ($p = 0.203$, paired t-test). The spike rate was compared between the 3 x 1 mins flow stoppages and the 3 x 1 mins prior to each flow stoppage.

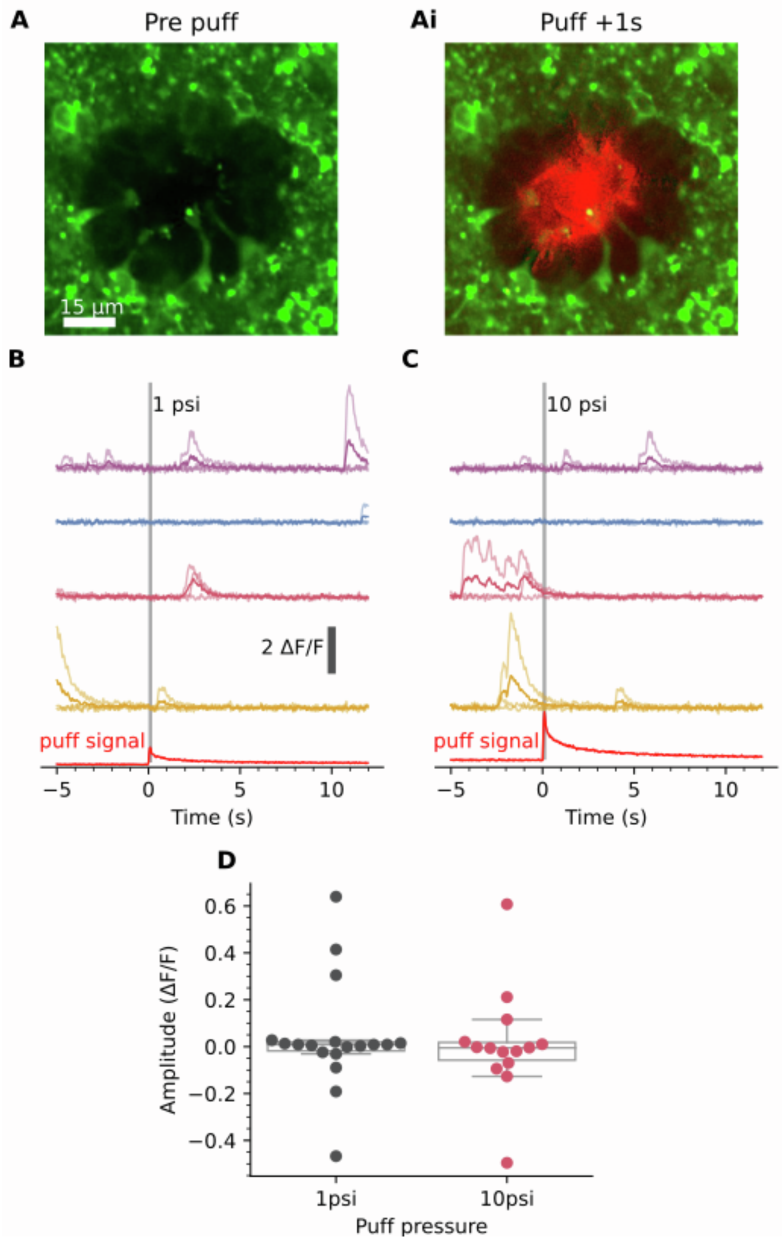

Figure S3 aCSF flow down the central canal does not modulate CSFcN activity, related to Figure 4 and STAR Methods. A the central canal before and 1s after (Ai) an ACSF puff down the central canal. The puffing pipette contained Alexa 594 to confirm delivery. **B** Activity of the 4 cells indicated by arrows in A with 3 repeats shown in light colour with the mean in dark for 1 psi. **C** same as B but with a 10 psi stimulus. **D** The amplitude of activity evoked by puffs applied over the central canal. The mean ΔF/F in the second before the puff was subtracted from the mean ΔF/F in the second after (n = 18 for 1psi, n=14 for 10psi, N=3). Outliers are due to spontaneous activity either before (negative values) or after puff (positive values).
